# Anterior Traumatic Dental Injuries in East Iranian School Children: Prevalence and Risk Factors

**Published:** 2014-12-24

**Authors:** Armita Rouhani, Taraneh Movahhed, Jamileh Ghoddusi, Yones Mohiti, Elham Banihashemi, Majid Akbari

**Affiliations:** a * Dental Research Center, Dental School, Mashhad University of Medical Sciences, Mashhad, Iran; *; b * Department of Oral and Maxillofacial Radiology, Babol University of Medical Sciences, Babol, Iran; *; c * Dental Student, Mashhad University of Medical Sciences, Mashhad, Iran*

**Keywords:** Dental Trauma, Iran, Permanent Dentition, Prevalence, Primary School, Traumatic Dental Injuries

## Abstract

**Introduction:** The aim of this cross-sectional study was to determine the prevalence and etiology of traumatic dental injuries (TDI) in school children of the Northeast of Iran. The type of involved teeth, the place of injury and treatment quality as well as the relationship between TDI and anatomic predisposing factors such as overjet and lip coverage were evaluated. **Methods and Materials:** A total of 778 school children were clinically examined for signs of trauma to their permanent teeth and the amount of overjet and lip coverage were also recorded. A questionnaire containing demographic data of participants and history of the dental trauma was given to the children’s parents. The data were analyzed using the chi-square and Mann-Whitney tests. **Results: **One hundred and seventy eight (22.9%) children had a history of previous trauma to their permanent teeth. There was a significant difference between boys and girls (*P*=0.017). A total of 46.1% of children had experienced luxation injuries of permanent teeth, 37% had crown fractures, and 16.9% experienced avulsion of anterior teeth. Maxillary central incisors were the most commonly affected teeth (84%). There was a significant relationship between TDI and overjet (*P*=0.02) in permanent teeth. On the other hand, there was no statistically significant relationship between TDI and lip coverage. The most common cause of TDI was falling over (42.9%) followed by fighting (34%). The majority of traumas happened at home (46.8%) and school (29.9%). Sixty two (39.7%) children with TDI did not receive any dental or medical care after injury. **Conclusion: **The prevalence of dental trauma in school children in Iran was rather high (22.9%); the most common type of trauma to the permanent teeth was luxation injuries.

## Introduction

Traumatic dental injuries (TDI) are prevalent and lead to a stressful situation for the children and their parents [1]. Majority of TDI cases occur in 7-10 year old children and the cause of trauma to anterior permanent teeth in most cases was reported to be falls, collision to people or objects, driving accidents, sports and violence [2].

These injuries commonly involve anterior maxillary teeth more than mandibular [3]. Moreover, most of TDI cases involve anterior teeth which might lead to difficulties in speaking, restrictions in biting and being ashamed of teeth appearance [1]. Beside the probable psychoemotional effects of this situation on children and their parents [4], malocclusions may occur in a short time due to the loss of proximal and incisal contacts [5].

The prevalence of TDI is relatively high in many countries [5]. In Iran, Navabazam and Farahani [6] have reported a prevalence of 27.56% for TDI to the anterior teeth in Yazd. Furthermore, treatment of TDIs in primary and permanent dentition is sometimes neglected [7, 8]. The aim of current study was to determine the prevalence of TDI and its associated factors among primary school children, in Mashhad, Iran.

## Materials and Methods

This cross-sectional study was performed on primary school children in Mashhad during the period of March 2012 to May 2012 and was approved by the Ethical Committee of Mashhad University of Medical Science (Grant no: 901054).

For sample size calculation three regions were randomly selected from 7 districts in Mashhad. Afterwards, one school for girls and one school for boys and 3 classes from each school were randomly selected. With prior arrangement with the school authorities in charge, appointments were done.

A total of 778 children with 6-12 years of age were included in this study. Two dentists clinically examined all subjects for signs of previous TDI to their permanent teeth. The clinical examination evaluated persistent traces of dental trauma, involved teeth and the type of injury. Previous dental trauma was categorized as *i)* crown fracture, *ii) *luxation, and *iii) *avulsion. Root fractures were not recorded in this study because dental radiographs are not considered appropriate for their diagnosis.

Children were examined for anterior TDI. Before the clinical examination, wet gauze pads were used to clean the tooth surfaces and a visual examination with a plane dental mirror was conducted. Lip competency and the amount of overjet were also recorded with a sterile gauge. In order to measure the overjet, the distance between the edge of maxillary central incisors and buccal surface of mandibular incisors was recorded in anterior-posterior axis when jaws were closed. Normal overjet was considered as 0-3 mm and the increased overjet was classified into 3-6 mm and more than 6 mm. Lip competency was recorded when the lips were able to contact each other without strain at rest position of mandible and if interlabial distance at the rest position was more than 3-4 mm, it was considered as lip incompetency [9].

A predesigned standardized questionnaire was also given to the parents of the children to gather further specific information concerning the dental trauma. The following data was recorded; the cause of the trauma (classified into fall, sport, road accident and fight), location of trauma (home, school, playground or other places), the age of child at the time of trauma and if the child received any dental treatment following the TDI, and the time between trauma and treatment. An informed consent was obtained from the parents.

The chi-square and Mann-Whitney tests were used to analysis the data with SPSS software version 13.0 (SPSS, Chicago, IL, USA) with the level of significance set at 0.05.

## Results

A total of 778 children were evaluated in this study including 349 (45%) girls and 429 (55%) boys. The mean age of the children was 10.3±2.1 ranging from 7 to 12 years old.

One hundred and seventy eight of the children showed signs of previous TDI which indicates a prevalence of 22.9% in 

the population (26.1% boys and 18.9% girls). There was a significant difference between injured boys and girls with most of the TDI cases happening to boys (*P*=0.01).

The type of injury in 46.1% of children was luxation, 37% crown fractures, and 16.9% avulsion. There was no statistically significant correlation between the type of injury and sex (*P*=0.95) or age (*P*=0.42).

Maxillary central incisors were the most commonly affected teeth (84%) followed by mandibular central incisors (7.5%), maxillary lateral incisors (4.5%), and maxillary canines (3%).

Statistical analysis of data showed that there was a significant relationship between TDI and overjet in permanent dentition (*P*=0.02). On the other hand, there was no significant relationship between TDI and lip (in)competency (*P*=0.98) (Table 1). 

Among 178 children with histories of TDI, a total of 156 questionnaires were obtained (response rate=87.6%). Further analysis was done on their data. The most common cause of TDI was fall (42.9%) followed by fight (34%). The majority of the TDI happened at home (46.8%) and then school (29.9%). Results are detailed in Table 2.

Sixty two (39.7%) children with TDI, had never been treated by a professional; while 49 (31.4%) cases referred to hospital or dental clinics and 45 (28.9%) to private offices for further evaluations. Forty six (48.9%) children with TDI were visited by a dentist on the accident day, 13 (13.8%) were visited within 1 to 7 day after accident, 9 (9.6%) within the first month and 26 (27.7%) sought for treatment when pain and discomfort occurred. There was no relation between the level of parents education and the rate of visiting the dentist (*P*=0.45).

## Discussion

Total prevalence of TDIs among the primary school children in this study was evaluated as 22.9% which is relatively high in comparison with previous studies [10, 11]. This prevalence has been reported differently in various studies [12, 13] which might be due to the different designs, various diagnostic criteria, restrict age ranges and behavioral and geographic differences between various sites of researches [14]. Occurrence of TDI among American and European teenagers was reported in a range of 15-23% and 23-35% [15, 16], respectively while this prevalence was reported as 4-35% in Asian juveniles [11]. Prevalence of anterior TDI was reported in a range of 6-19% in Asia and Pacific Ocean region [10]. In some studies which were performed in Iran, the prevalence of TDI differed from 8.96% in Tehran [17] to 27.56% in Yazd [6]. 

**Table 1:** Prevalence and the correlation between traumatic dental injuries (TDI), lip coverage and overjet

		**Presence of TDI, N (%)**	**Absence of TDI, N (%)**	**Total, N (%)**
**Lip coverage**	**Competent**	170 (23.4)	555 (76.6)	725 (93.1)
	**Incompetent**	12 (22.7)	41 (77.3)	53 (6.9)
**Overjet**	**<3 mm**	128 (20.7)	490 (79.3)	618 (79.5)
	**3-6 mm**	46 (33.3)	92 (66.7)	138 (17.8)
	**≥ 6 mm**	8 (36.3)	14 (63.7)	22 (2.7)

**Table 2:** Distribution of traumatic dental injuries (TDI) in relation with location and cause

**Cause/location of TDI**	**N (%)**
**Fall **	67 (42.9)
**Fight **	53 (34)
**Sport **	9 (5.8)
**Accident **	7 (4.5)
**Cycling **	8 (5.1)
**Others **	12 (7.7)
**Home **	74 (47.4)
**School **	46 (29.5)
**Playground **	10 (6.4)
**Others **	26 (16.7)

Total prevalence of TDIs among the primary school children in this study was evaluated as 22.9% which is relatively high in comparison with previous studies [10, 11]. This prevalence has been reported differently in various studies [12, 13] which might be due to different design of studies, various diagnostic criteria, restrict age ranges and behavioral and geographic differences between various sites of researches [14]. Occurrence of TDI among American and European teenagers was reported in a range of 15-23% and 23-35% [15, 16], respectively while this prevalence was reported as 4-35% in Asian juveniles [11]. Prevalence of anterior TDI was reported in a range of 6-19% in Asia and Pacific Ocean region [10]. In some studies which were performed in Iran, the prevalence of TDI differed from 8.96% in Tehran [17] to 27.56% in Yazd [6].

In the current study the most common type of injury among the traumatized children was luxation (46.1%). In another national study, the most prevalent dental injuries were enamel-dentin fractures [17, 18]. In the study by Gupta *et al.* [2], and Rajab *et al. *[13], enamel-dentin fractures were also the most prevalent dental injuries. In a study in which prevalence of TDI was assessed in school children in Brazil, enamel fractures (66%) were the most common type of dental traumas followed by enamel-dentine fractures (27%) [19]. A good explanation about the numerical preference of luxation injuries in the present study can be the lower age of children and consequently more spongy character of alveolar bone.

Maxillary central incisors were the most common traumatized teeth in our study (84%) similar to results of the previous studies by Navabazam and Farahani [6], Ansari and Mobini [17], Ghahramani *et al. *[18], Rajab *et al*. [13], Altun *et al.* [20], David *et al*. [21] and Ferreira *et al*. [22]. In addition in a study conducted in India it was reported that 95% of traumatic dental injuries involved maxillary anterior teeth [2].

In regards to the relation between prevalence of TDIs, overjet and type of lip coverage, a statistically significant correlation was reported between TDIs and overjet in this study (*P*=0.02). So it can be concluded that overjet more than 3 mm may cause the child being more susceptible to TDIs but there was not a statistically significant relationship between the type of lip coverage and occurrence of TDIs. In the study by Rajab *et al.* [13] it was concluded that lip incompetency as well as increased overjet (more than 3 mm) are both related to TDIs. In addition, Francisco *et al.* [19] revealed that occurrence of traumatic dental injuries in children with overjet more than 3 mm is 1.78 more than children with 3 mm overjet and children with incompetent lips are also two times more susceptible to dental injuries compared to children with competent lip. This enhanced risk of dental trauma in children with increased overjet, has also been reported in other studies [2, 3, 20].

In our study, the major cause of dental traumas was falling over (42.9%) which was similar to the prevalence reported by Navabazam and Farahani [6], Ghahramani *et al. *[18], Yassen *et al.* [23] and Bhayya and Shyagali [12]. Moreover, boys experience TDI significantly more than girls (*P*<0.05), which is along with the results of several other studies [2, 3, 20, 21] and might be due to the more enthusiasm of boys in to violent plays and activities.

It was also revealed that prevalence of TDI enhanced significantly with age (*P*=0.02). However, it does not mean that elderly children are more susceptible to dental injuries but it shows a cumulative characteristic of TDI records in time. This was also observed in some other studies in which dental injuries became more prevalent with age [2, 22].

In the present study, 39.7% of children who had a history of dental trauma didn’t refer to dental professionals in order to obtain professional assessment which might be due to the low level of parental awareness regarding the importance of management of traumatized teeth or their low socioeconomic conditions. Similarly in the study by Gupta *et al.* [2], 97.7% of traumatized teeth didn’t receive a professional evaluation by dentists. In another similar study, only 1.68% of traumatized cases were treated [24].

Although in this study the sample size was relatively high (778 children), further studies with grater sample size are recommended.

## Conclusion

Due to the relatively high prevalence of traumatic dental injuries (TDI) in primary school children assessed in this study, holding proper educational programs in regard to enhance the level of general knowledge about prevention and managing these injuries seems necessary.
